# Scale-up and large-scale production of *Tetraselmis* sp. CTP4 (Chlorophyta) for CO_2_ mitigation: from an agar plate to 100-m^3^ industrial photobioreactors

**DOI:** 10.1038/s41598-018-23340-3

**Published:** 2018-03-23

**Authors:** Hugo Pereira, Jaime Páramo, Joana Silva, Ana Marques, Ana Barros, Dinis Maurício, Tamára Santos, Peter Schulze, Raúl Barros, Luísa Gouveia, Luísa Barreira, João Varela

**Affiliations:** 1 CCMAR - Centre of Marine Sciences, University of Algarve, Gambelas, 8005-139 Faro, Portugal; 2 CMP - Cimentos Maceira e Pataias, ALGAFARM – Unidade de Produção de Microalgas, 2445 – 411, Pataias, Portugal; 3 Faculty of Biosciences and Aquaculture, Nord University, 8049 Bodø, Norway; 4 CIMA - Centro de Investigação Marinha e Ambiental, University of Algarve, Gambelas, 8005-139 Faro, Portugal; 5 LNEG - Laboratório Nacional de Energia e Geologia, I.P./Bioenergy Unit, Estrada do Paço do Lumiar 22, 1649-038 Lisbon, Portugal

## Abstract

Industrial production of novel microalgal isolates is key to improving the current portfolio of available strains that are able to grow in large-scale production systems for different biotechnological applications, including carbon mitigation. In this context, *Tetraselmis* sp. CTP4 was successfully scaled up from an agar plate to 35- and 100-m^3^ industrial scale tubular photobioreactors (PBR). Growth was performed semi-continuously for 60 days in the autumn-winter season (17^th^ October – 14^th^ December). Optimisation of tubular PBR operations showed that improved productivities were obtained at a culture velocity of 0.65–1.35 m s^−1^ and a pH set-point for CO_2_ injection of 8.0. Highest volumetric (0.08 ± 0.01 g L^−1^ d^−1^) and areal (20.3 ± 3.2 g m^−2^ d^−1^) biomass productivities were attained in the 100-m^3^ PBR compared to those of the 35-m^3^ PBR (0.05 ± 0.02 g L^−1^ d^−1^ and 13.5 ± 4.3 g m^−2^ d^−1^, respectively). Lipid contents were similar in both PBRs (9–10% of ash free dry weight). CO_2_ sequestration was followed in the 100-m^3^ PBR, revealing a mean CO_2_ mitigation efficiency of 65% and a biomass to carbon ratio of 1.80. *Tetraselmis* sp. CTP4 is thus a robust candidate for industrial-scale production with promising biomass productivities and photosynthetic efficiencies up to 3.5% of total solar irradiance.

## Introduction

Most microalgae are unicellular photosynthetic organisms that through photosynthesis and several metabolic pathways convert inorganic carbon (CO_2_) into organic carbon in the form of proteins, lipids, carbohydrates and nucleic acids. Therefore, the industrial production of microalgal biomass couples the mitigation of CO_2_ with the production of biomolecules that can be purified or upgraded into bioproducts important for different biotechnological applications (e.g. food, feed, pharmaceuticals and biofuels). Although several microalgae ventures have been established in recent years^1^, the implementation of industrial biomass production is still at an infant stage^2^. Nevertheless, mass culture of microalgal biomass is currently considered as one of the most promising approaches to manufacturing next-generation foods, feeds, and biofuels with the concomitant capture of CO_2_ from emitting industries and recycling nutrients from wastewaters^3,4^.

**Figure 1:**
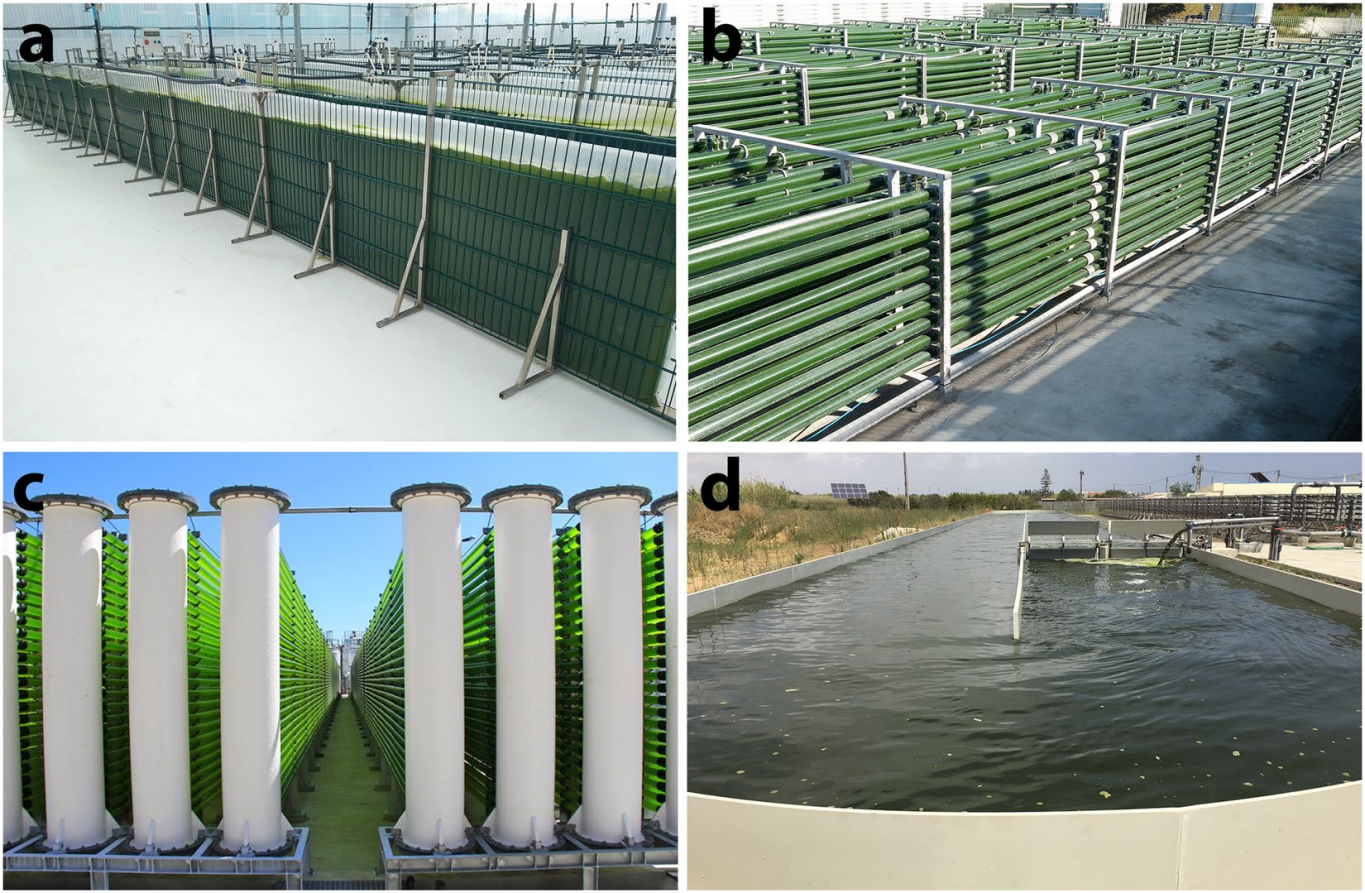
Different large-scale systems currently used for the industrial production of microalgal biomass: (**a**) 1-m^3^ Flat panel photobioreactor. (**b**) 2.5-m^3^ pilot-scale tubular photobioreactor. (**c**) 100-m^3^ industrial tubular photobioreactor. (**d**) 200-m^3^ raceway. Pictures depicted were kindly provided by CMP, Secil group, Pataias, Portugal (**a**–**c**) and Necton S.A., Belamandil-Olhão, Portugal (**d**).

Mass culture of microalgae can be achieved in open (e.g., open ponds or raceways) or closed (e.g., photobioreactors; PBR) production systems (Fig. 1). Open ponds are the system chosen by most companies producing microalgae at an industrial scale due to the low capital and operational costs^5–8^. However, as cultures are directly exposed to the atmosphere, the water and CO_2_ losses and the probability of contamination are the main hindrances of open production systems^7^. In addition, the strict control of temperature and other culture parameters required to grow sensitive strains (e.g. diatoms) is rather challenging^9^. On the other hand, closed systems display lower CO_2_ and water losses, reduce the probability of contamination and allow a tighter control of growth conditions. This allows the cultivation of most microalgal strains^5,10,11^ with higher areal and volumetric biomass productivities^5,7^.

In order to meet the full potential of microalgal biomass, the selection of robust and fast-growing strains is crucial to develop feedstocks that can effectively grow in large-scale industrial facilities^12,13^. Recently, *Tetraselmis* sp. CTP4 was isolated and characterised as a robust, euryhaline, lipid-rich microalga able to grow both in standard growth media, as well as in urban wastewater effluents^14,15^. Apart from its high potential for bioremediation, *Tetraselmis* sp. CTP4 presents promising features as compared to common microalgal feedstocks. The biomass of this microalga can be recovered through natural cell sedimentation, decreasing the total culture volume down to 20% within 6 hours^14^. This property is essential to significantly decrease harvesting costs, one of the most costly steps of culturing and retrieving microalgae from an aqueous growth medium^16^.

Because of the high potential of *Tetraselmis* sp. CTP4 for different biotechnological applications, the present work describes the scale-up procedure used to reach industrial production. To enhance the biomass production, the culture velocity and pH set point for CO_2_ injection were tested and optimized in a pilot-scale tubular PBR. To the authors’ knowledge, this is the first report addressing CO_2_ mitigation as well as biomass and lipid productivities of microalgae cultures grown semi-continuously in an industrial-scale tubular PBR production system.

## Results

### Optimization of culture velocity and pH set point

**Figure 2:**
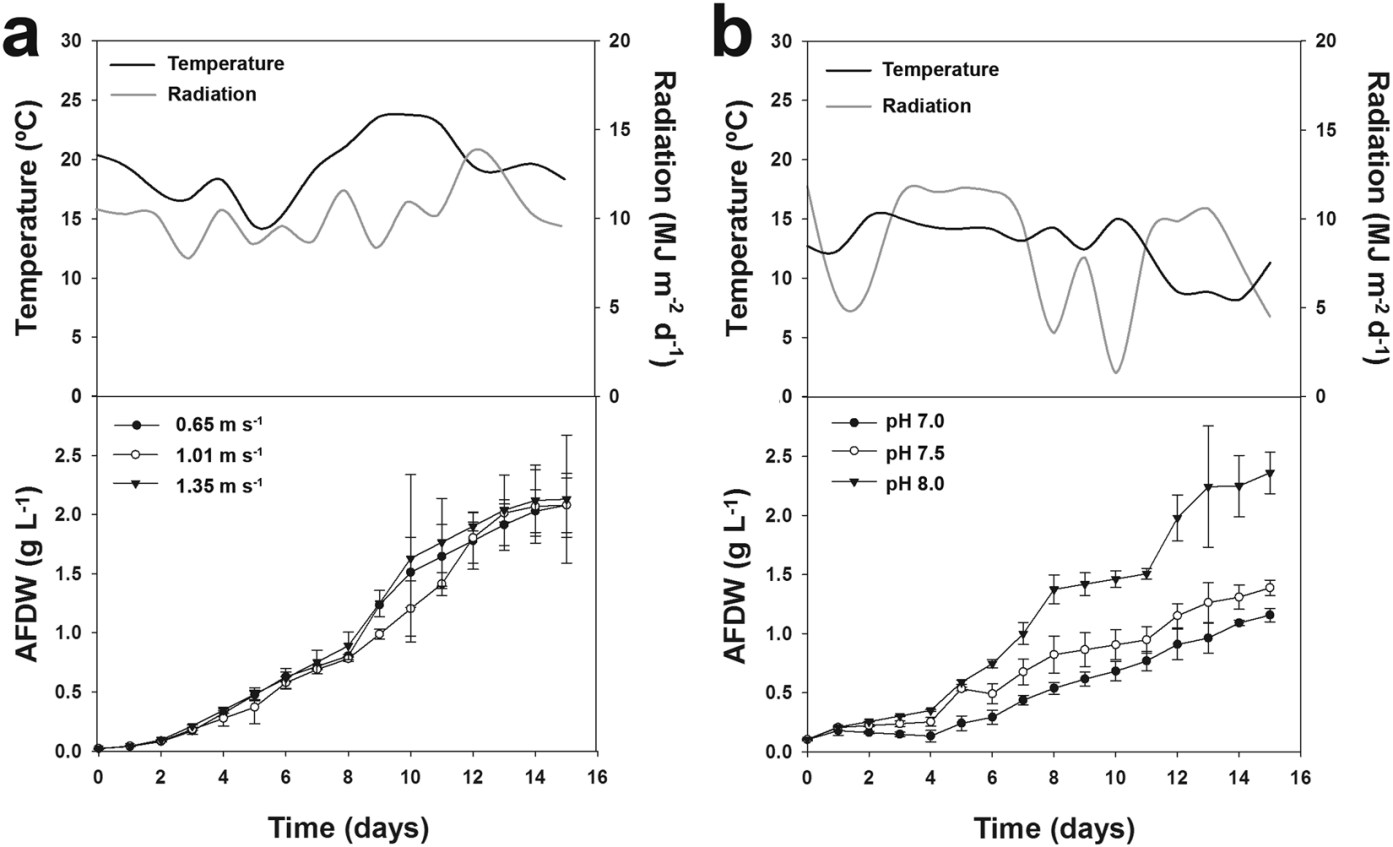
Optimization of tubular photobioreactor operation in pilot-scale production systems. *Tetraselmis* sp. CTP4 growth in 2.5-m^3^ tubular photobioreactors. (**a**) Culture velocity. (**b**) pH set point for CO_2_ injection.

In a first experiment, the culture velocity was tested in 2.5 m^3^ pilot-scale tubular PBR using three different culture velocities: 0.65, 1.01 and 1.35 m s^−1^. The radiation during the trial was 10.3 ± 1.7 MJ m^−2^ d^−1^, while the temperature was 19.4 ± 2.9 °C (Fig. 2a). Cultures under all conditions displayed similar growth patterns, without significant differences among them (*p* > 0.05), reaching the late exponential phase at day 13 and a final ash free dry weight (AFDW) of approximately 2.1 g L^−1^. The same pattern was observed for the volumetric and areal biomass productivities (0.14–0.15 g L^−1^ d^−1^ and 12.9–13.6 g m^−2^ d^−1^, respectively), where no significant differences were observed (*p* > 0.05) under all velocities tested (Table 1). The same was found for the maximum biomass productivity under all conditions (0.36–0.43 g L^−1^ d^−1^ and 34.7–39.1 g m^−2^ d^−1^).

**Table 1:** Volumetric and areal biomass productivities presented in ash free dry weight of batch cultures grown in 2.5-m^3^ outdoor tubular photobioreactors, using different culture velocities and pH set points for CO_2_ injection. Different letters indicate significant differences within each parameter tested.

PBR	Volumetric productivity		Areal productivity	
	Total	Max	Total	Max
	g L^−1^ d^−1^	g L^−1^ d^−1^	g m^−2^ d^−1^	g m^−2^ d^−1^
***Culture velocity (m s*** ^***−1***^ **)**				
0.65	0.14 ± 0.02^a^	0.43 ± 0.15^a^	12.9 ± 1.44^a^	39.1 ± 9.19^a^
1.01	0.15 ± 0.01^a^	0.39 ± 0.09^a^	13.6 ± 0.52^a^	35.4 ± 5.12^a^
1.35	0.15 ± 0.02^a^	0.36 ± 0.10^a^	13.6 ± 2.01^a^	34.7 ± 8.28^a^
***pH set point***				
7.0	0.07 ± 0.01^a^	0.14 ± 0.02^a^	7.8 ± 0.39^a^	13.1 ± 1.85^a^
7.5	0.08 ± 0.01^a^	0.20 ± 0.05^a^	9.4 ± 0.44^a^	16.9 ± 3.28^a^
8.0	0.15 ± 0.02^b^	0.37 ± 0.04^b^	15.9 ± 1.19^b^	34.1 ± 8.90^b^

Afterwards, a trial was performed in the same PBRs (2.5 m^3^) to assess the effect of different pH set points on CO_2_ injection (Fig. 2b). The temperature (12.7 ± 3.3 °C) and daily radiation (8.3 ± 3.3 MJ m^−2^ d^−1^) observed during this trial were lower than those of the previous experiment (Fig. 2a). Interestingly, the different tested pH set points affected the growth of *Tetraselmis* sp. CTP4, displaying significant differences between cultures maintained at pH 8 compared to pH 7.5 and 7.0 (*p* < 0.05). Accordingly, best growth was obtained at a pH set point of 8.0, with higher volumetric and areal biomass productivities (0.15 g L^−1^ d^−1^ and 15.9 g m^−2^ d^−1^, respectively). Cultures at pH 7.5 (0.08 g L^−1^ d^−1^ and 9.4 g m^−2^ d^−1^) and neutral pH (7.0) displayed the lowest growth performances (0.07 g L^−1^ d^−1^ and 7.8 g m^−2^ d^−1^). Consequently, faster growth (Fig. 2b) and maximum biomass productivity (Table 1) were achieved at pH 8.0 (0.37 g L^−1^ d^−1^ and 34.1 g m^−2^ d^−1^), compared to that of pH 7.5 (0.20 g L^−1^ d^−1^ and 16.9 g m^−2^ d^−1^) and 7.0 (0.14 g L^−1^ d^−1^ and 13.1 g m^−2^ d^−1^).

### Growth in industrial scale photobioreactors

**Figure 3:**
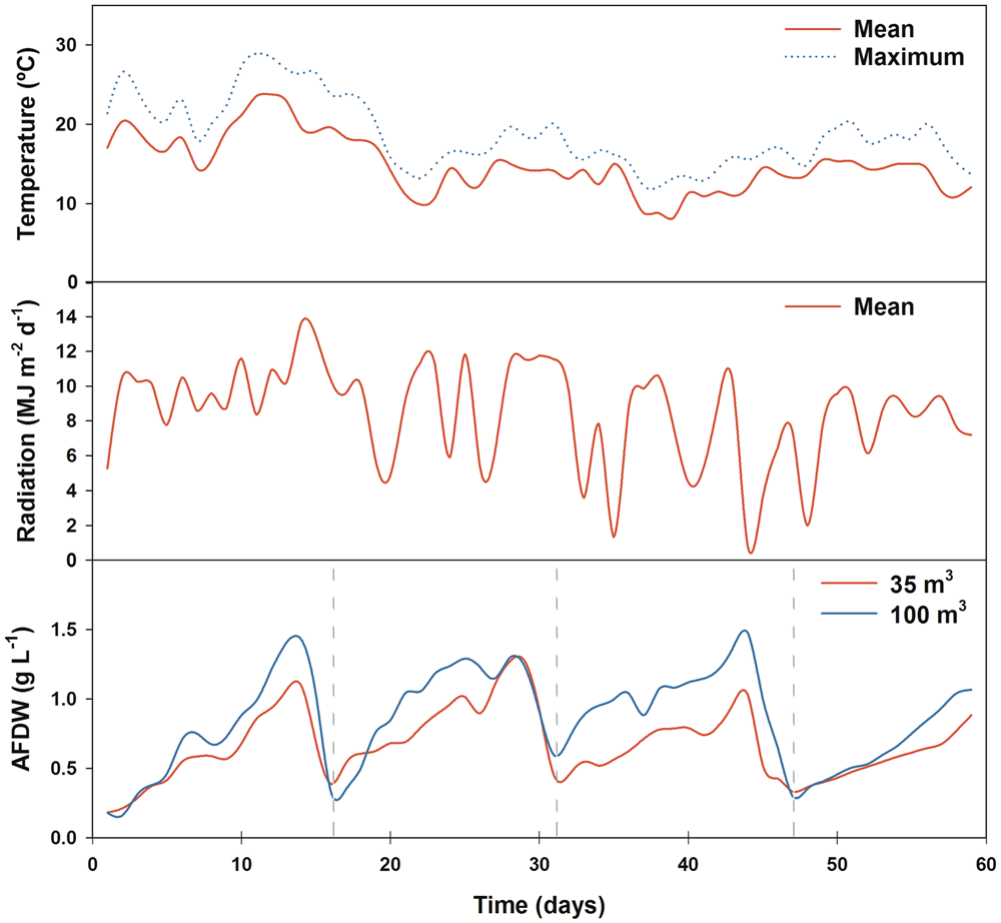
Mean and maximum temperature and radiation registered during the growth of *Tetraselmis* sp. CTP4 in 35- and 100-m^3^ industrial tubular photobioreactors grown semi-continuously. Cultures were harvested every 13–14 days for approximately 60 days, between 17^th^ October and 15^th^ December. Dashed grey line marks the start of the following growth period.

After the optimization of the culture conditions, cells were grown semi-continuously in 35- and 100-m^3^ industrial tubular PBR for approximately 60 days (Fig. 3) and harvested four times, every 13–14 days. Experiments were carried out (17^th^ October – 14^th^ December) in a non-optimal season. In fact, the second half of this time range partially overlaps with the months when temperature and irradiance are lowest in the northern hemisphere. Ambient temperature decreased from 19.2 ± 2.9 °C during 17–30^th^ October to 12.9 ± 2.5 °C between the 2^nd^ November − 14^th^ December. The same pattern was observed for the daily radiation, decreasing from 9.7 ± 1.9 MJ m^−2^ d^−1^ during the first 15 days to 7.9 ± 2.9 MJ m^−2^ d^−1^ due to higher cloud cover. Both PBRs were inoculated at a concentration of ~0.2 g L^−1^. Notably, the 100 m^3^-system displayed on average higher biomass concentrations than the 35 m^3^ system (*p* < 0.05) with average concentrations of 1 g L^−1^ and 0.8 g L^−1^, respectively. As compared to the 35-m^3^ system, the 100-m^3^ PBR registered higher volumetric (0.08 ± 0.01 vs. 0.05 ± 0.02 g L^−1^ d^−1^) and areal (20.3 ± 3.2 vs. 13.5 ± 4.3 g m^−2^ d^−1^) biomass productivities (*p* < 0.05; Table 2) as well as photosynthetic efficiencies (PEs; 3.35 ± 0.19 vs. 2.38 ± 0.27%; *p* < 0.05). In addition, the areal productivities were statistically higher during the first 30 days (35 m^3^: 17.1 ± 1.9 g m^−2^ d^−1^; 100 m^3^: 22.4 ± 3.5 g m^−2^ d^−1^) as compared to the last 30 days (35 m^3^: 9.9 ± 0.6 g m^−2^ d^−1^; 100 m^3^: 18.2 ± 0.4 g m^−2^ d^−1^) in both PBRs. This result can be explained by the lower temperatures and radiation observed on site.

**Table 2:** Volumetric and areal biomass productivities of *Tetraselmis* sp. CTP4 grown semi-continuously in 35- and 100-m^3^ tubular photobioreactors (PBRs) presented in ash free dry weight. The photosynthetic efficiency (PE) obtained in the different growth periods is also presented. Using a semi-continuous growth system, four different culture periods were established throughout the growth trial (17^th^ Oct–15^th^ Dec). Different letters indicate significant differences in productivity and PE between PBRs.

PBR	Biomass productivity				PE
	Total	Max	Total	Max	(%)
	g L^−1^ d^−1^	g L^−1^ d^−1^	g m^−2^ d^−1^	g m^−2^ d^−1^	
**3** ***5 m*** ^**3**^					
17^th^ − 30^th^ Oct	0.07	0.18	18.4	46.8	2.62
2^nd^ − 14^th^ Nov	0.06	0.15	15.8	39.1	2.59
17^th^ − 29^th^ Nov	0.04	0.15	10.3	41.7	2.20
1^st^ − 14^th^ Dec	0.04	0.10	9.5	27.1	2.09
Mean	0.05 ± 0.02^a^	0.15 ± 0.03^a^	13.5 ± 4.3^a^	38.7 ± 8.4^a^	2.38 ± 0.27^a^
***100 m*** ^***3***^					
17^th^ − 30^th^ Oct	0.10	0.19	24.9	42.4	3.54
2^nd^ − 14^th^ Nov	0.08	0.20	20.0	42.8	3.28
17^th^ − 29^th^ Nov	0.07	0.18	18.5	40.4	3.46
1^st^ − 14^th^ Dec	0.07	0.10	18.0	23.1	3.11
Mean	0.08 ± 0.01^b^	0.17 ± 0.05^a^	20.3 ± 3.2^b^	37.2 ± 9.4^a^	3.35 ± 0.19^b^

Taking into account the meteorological weather data and normalising the areal and volumetric productivities for both PBRs, a strong positive correlation between productivity and supplied irradiation (*r* = 0.97; *p* < 0.05) and temperature (*r* = 0.89; *p* < 0.05) was found. Temperature was also found to affect the PE, decreasing by 15% when this parameter dropped below 15 °C. This indicates that growth performance of this strain in both PBRs was strongly affected by light and temperature and that CTP4 tends to grow better at temperatures above 15 °C. The maximum volumetric and areal productivities observed in the 35- (0.15 ± 0.03 g L^−1^ d^−1^ and 38.7 ± 8.4 g m^−2^ d^−1^) and 100-m^3^ (0.17 ± 0.05 g L^−1^ d^−1^ and 37.2 ± 9.4 g m^−2^ d^−1^) PBRs were similar (*p* > 0.05), reaching the double of the average productivities in most growth periods.

**Figure 4:**
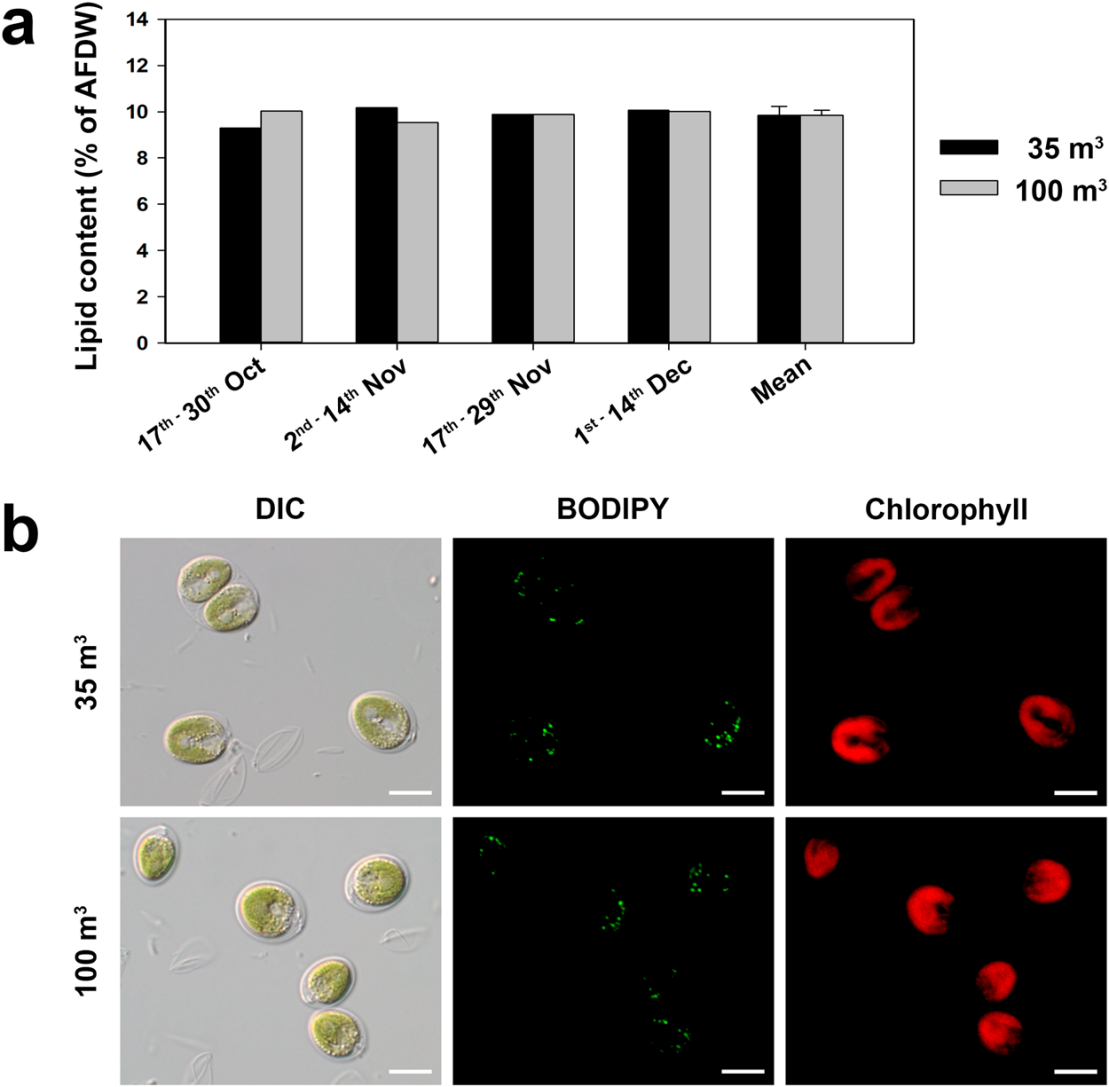
Lipid content and fluorescence microscopy of *Tetraselmis* sp. CTP4 grown semi-continuously in tubular photobioreactors. (**a**) Lipid content of cultures grown industrially in four different growth periods and mean value obtained in the overall experiment. (**b**) Brightfield and fluorescence microscopy of cultures grown in the in the 35- and 100-m^3^ tubular photobioreactors. Depicted pictures show the differential interference contrast (DIC), as well as BODIPY 505/515 and chlorophyll fluorescence of *Tetraselmis* sp. CTP4 cells. Scale bar = 10 µm.

The volumetric and areal lipid productivities were about 10% of the respective biomass productivities, since the lipid content in the biomass produced throughout the four growth periods and in both PBRs was quite stable, averaging 9.9 ± 0.3% of AFDW (Fig. 4a). The results were confirmed by fluorescence microscopy of cells stained with BODIPY 505/515 (Fig. 4b). Overall, obtained results revealed that the lipid content was not significantly affected by the volume of the PBR, temperature or light intensity (*p* > 0.05).

### CO_2_ sequestration

The capacity of *Tetraselmis* sp. CTP4 to mitigate CO_2_ was investigated in the 100-m^3^ PBR for 30 days (17^th^ Oct – 17^th^ Nov). The mass balance of CO_2_, considering the CO_2_ that enters the system and the CO_2_ exhausted from the PBR, was related with the C content of the biomass (determined by elemental analysis). In agreement with the elemental analysis of the biomass that showed mass contents of 49.1% C, 7.84% H and 5.80% N, the following approximate stoichiometry can be used to describe biomass formation from CO_2_ and nitrate:

1 $$1.101{{\rm{CO}}}_{2}+0.101{{{\rm{NO}}}_{3}}^{-}+1.008{{\rm{H}}}_{2}{\rm{O}}\to {{\rm{CH}}}_{1.914}{{\rm{O}}}_{0.568}{{\rm{N}}}_{0.101}+1.321{{\rm{O}}}_{2}+0.101{{{\rm{HCO}}}_{3}}^{-}$$

This equation shows that 1.80 g of CO_2_ are consumed for the formation of 1.0 g of ash free algal biomass. Accordingly, the CO_2_ mass balance in the 100-m^3^ PBR was performed by quantifying the volume of injected CO_2_ (99.99%), its content in the air used for degassing the culture (0.04%), and the CO_2_ content of the exhaust gas (0.3–0.5%). Even though the volumetric flow of the later stream was not accurately measured due to operational impracticability of placing a rotameter in the exhaust section of the PBR, it can be assumed that its molar flow will be quite close to that of the compressed air, because it is two orders of magnitude higher than pure CO_2_ injection. Our calculations (Supplementary data) show that 60–75% of the CO_2_ introduced in the PBR is taken up by the culture, while 25–40% of the CO_2_ is exhausted from the PBR to the atmosphere. In summary, a total of ~535 kg of CO_2_ were consumed to produce ~296 Kg biomass in the 100-m^3^ PBR during a 60-day operation.

### Season comparison using an Algem^®^ photobioreactor

**Figure 5:**
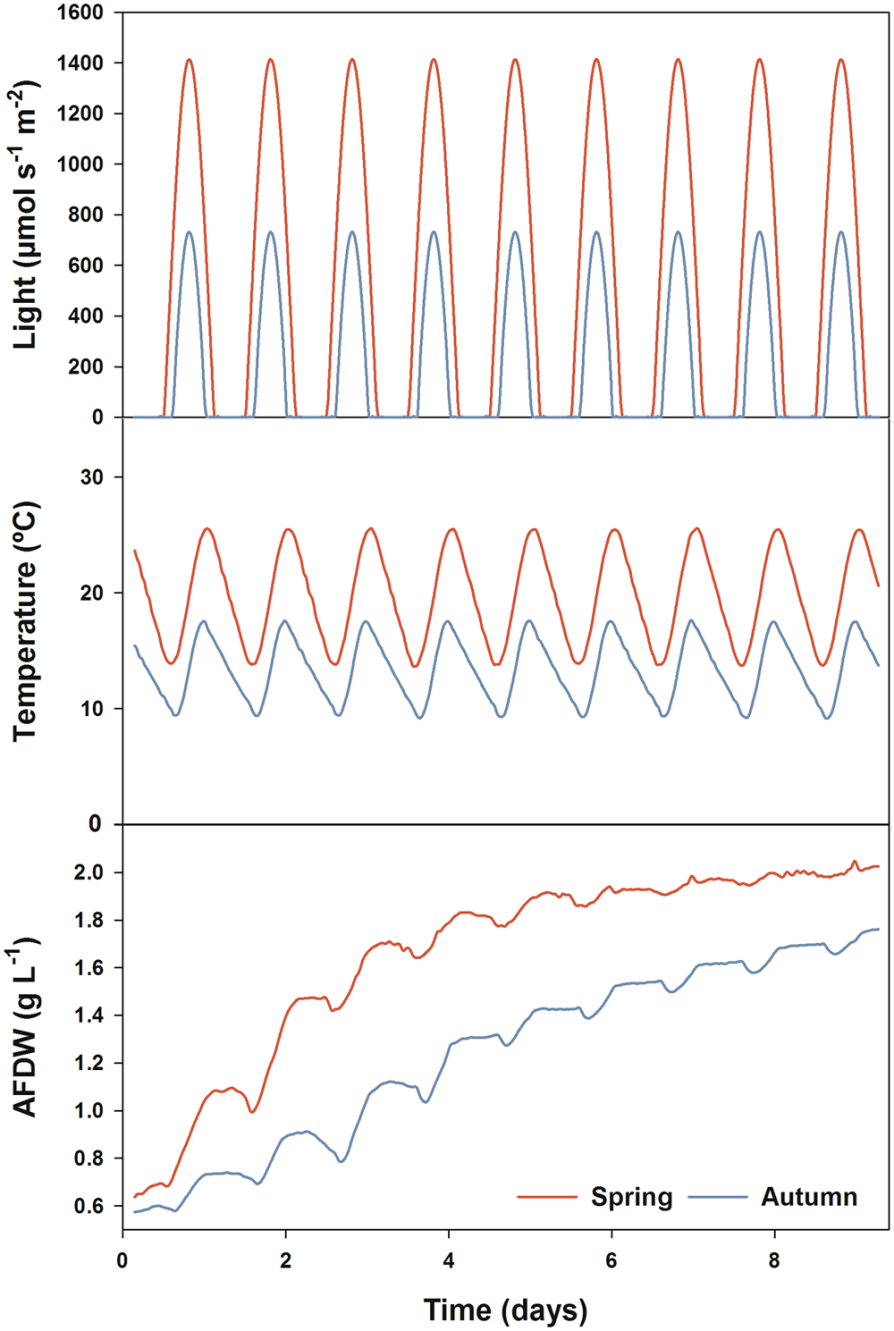
Season comparison assay of *Tetraselmis* sp. CTP4 in Algem^®^ photobioreactors. Growth curves of *Tetraselmis* sp. CTP4 using an Algem^®^ photobioreactor simulating the conditions of growth in Spring and Autumn seasons on the West coast of Portugal.

A season comparison assay was performed using an Algem^®^ PBR to simulate the Spring and Autumn seasons at the latitude and longitude of AlgaFarm using controlled artificial LED light. The main objective of the simulation was to estimate the growth potential of *Tetraselmis* sp. CTP4 under average abiotic conditions in order to expand the findings obtained outdoors. The Algem^®^ built-in software defines a maximum light intensity of 700 and 1400 µmol s^−1^ m^−2^ and a mean temperature of 12 and 20 °C for Autumn and Spring, respectively (Fig. 5). The growth conditions simulating Spring presented a higher growth rate, reaching the stationary phase in approximately 5 days with a final AFDW of 2.02 g L^−1^, and a biomass productivity of 0.25 g L^−1^ d^−1^. On the other hand, cultures grown in conditions simulating Autumn displayed a lower growth performance, reaching a final AFDW of 1.76 g L^−1^ and a biomass productivity of 0.12 g L^−1^ d^−1^ in the end of the assay (day 9). During day 1 and 9, the spring simulation yielded on average a significant higher biomass concentration (1.7 g L^−1^) as compared to the winter conditions (1.3 g L^−1^; *p* < 0.05*)*. These results suggest that the expected growth rate of cultures and effective CO_2_ mitigation has a marked seasonal dependence. This rate is expected to be twice as high in spring, when compared to its value in autumn (0.45 g CO_2_ L^−1^ d^−1^ vs 0.22 g CO_2_ L^−1^ d^−1^).

## Discussion

The trend of microalgal biotechnology towards a medium sized market requires studies about the optimization of industrial scale cultivation systems and novel strains to widen the current portfolio for maximal production efficiency^17^. The present study demonstrated the successful scale-up of the novel isolate *Tetraselmis* sp. CTP4 from an agar plate to a 100-m^3^ industrial tubular PBR within eight weeks. *Tetraselmis* sp. CTP4 is thus a promising candidate for mass production of bulk products due to high growth performance among various culturing systems and environmental conditions.

**Figure 6:**
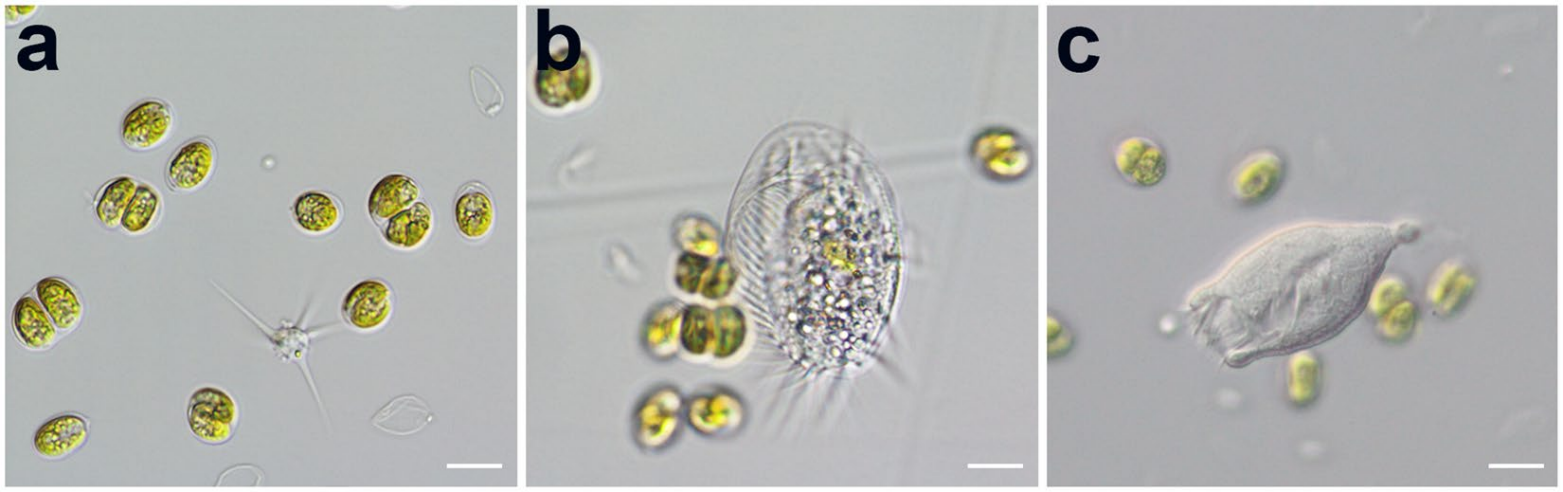
Different environmental contaminants detected in the course of the present work throughout the growth in industrial scale production systems. (**a**) *Amoeba radiosa*. (**b**) Unidentified ciliate. (**c**) *Vorticella* sp. Scale bar = 10 µm.

The pH of the culture medium is an essential parameter not only to obtain optimal growth, but it also determines the maximal amount of CO_2_ dissolved in the medium (carbon balance). *Tetraselmis* sp. CTP4 performed best at a slight alkaline pH of 8.0, a result similar to that reported by Khatoon *et al*.^18^ for microalgae of the same genus. However, as the response to pH fluctuations is species-dependent, optimal growth of *Tetraselmis suecica* was achieved by Moheimani^19^ at pH 7.0 and 7.5. The culture velocities tested were all suitable for growing *Tetraselmis* sp. CTP4 in tubular PBRs. This might be explained by the low radiation observed on site during this time period, since it has been observed that under low light conditions the mixing rates are less important for the final productivity^20^. The opposite is expected in the spring-summer season during which the importance of velocity might increase due to its effect on the overall light availability to cells when grown under higher radiation. In addition, the optimization of velocity suitable to the microalgal culture inside the production tubes is important to avoid biomass deposition while cells travel through the photic section of the PBR and increase CO_2_ availability^21,22^. Lower velocities can be used to reduce the energy costs in the production pipeline; however, this can lead to the formation of biofilm in the tubes, promoting light attenuation in the system (not observed in the present work). On the other hand, the use of higher culture velocities without lysing microalgal cells of interest can be important to manage and contain specific contaminations. This is particularly true for contaminants sensitive to the added turbulence and shear stress generated by faster velocities in the PBR^20^. An important factor for the successful implementation of a microalgal-based production pipeline is the proper management of predators and competing microalgae. Similarly, the euryhaline properties of *Tetraselmis* sp. CTP4 can be used to eliminate potential contaminants from large-scale production facilities by means of abrupt salinity shifts^14^, in particular if the contaminant does not have a cell wall or has reduced halotolerance. It is worth noting that during the scale-up procedure and all experimental trials, cultures of *Tetraselmis* sp. CTP4 remained monoalgal, i.e., no other microalgal species were detected. Although some common non-photosynthetic contaminants were observed they did not had a severe impact on productivity and did not take over the cultures (Fig. 6). In fact, all reactors were grown without any culture collapse in spite of the changing conditions of temperature and radiation. This is an important result, as some commonly used microalgal species (e.g. *Chlorella vulgaris* and *Haematococcus pluvialis*) are more susceptible to predators/parasites under industrial settings (e.g. *Chytridium* sp., *Amoeboaphelidium protococcarum* and *Vampirovibrio chlorellavorus*), which have a significant impact on culture viability and biomass productivity^23–25^.

Accordingly, semi-continuous growth of *Tetraselmis* sp. CTP4 at industrial scale was successfully achieved in both 35- and 100-m^3^ PBR for a 60-day period. As expected, the growth of cultures was higher in the first two growth periods (late October – early November), which resulted in higher biomass productivities. These results are in accordance with the data obtained from the statistical correlations obtained for both industrial PBR as well as in the Algem^®^ PBR, which strongly suggests that low temperatures (<15 °C) and radiation decreased the biomass productivities of this strain. However, *Tetraselmis* sp. CTP4 responded differently in the 35-m^3^ and 100-m^3^ PBRs, within the same time period. Interestingly, it was in the largest industrial PBR tested that higher productivities were consistently obtained. Volumetric biomass productivities in the industrial scale PBRs (35- and 100-m^3^) were lower than compared to the pilot-scale PBRs (2.5-m^3^), while the reverse trend was apparent for areal productivities. The reason for such difference relies on the high stocking density of the horizontal tubes in the industrial reactors. Although the lower light penetration into the industrial PBRs tubes at lower layers reduces the volumetric production and the final biomass concentration in the system, the considerably higher culture volume in the same area results in higher areal productivities.

The areal productivity registered in the first growth period (17^th^ – 30^th^ October) is similar to the productivity previously reported for other microalgal strains (e.g. *Phaeodactylum tricornutum* and *Nannochloropsis* sp.)^26^. In addition, the PEs of 3.35 ± 0.19% (100-m³) were high despite the shifting temperature and light regimes during the time period tested.

The average mitigation efficiency of 65% of the CO_2_ in the 100-m^3^ PBR was notable, considering the industrial size of system. This efficiency is considerably higher than previous reports that addressed CO_2_ mitigation using other microalgal strains and cultivation systems^27–30^. However, the values here reported are similar to those reported by Keffer & Kleinheinz^31^ using *Chlorella vulgaris* (74% carbon mitigation efficiency) fed with an elevated CO_2_ stream. Higher effective CO_2_ removal (82.5–99%) has been reported when *C. vulgaris* is grown using a laboratory-scale sequential PBR array^32^.

The biomass to carbon ratio of 1.80 obtained in this work is typical for non-stressed microalgae^8,12^. This ratio can be increased by higher amounts of lipids in biomass that display higher carbon content per unit mass (76–77%) than proteins (53%) or carbohydrates (40–44%)^33^. The values of the lipid content found in this work are in accordance with the data previously reported for *Tetraselmis* sp. CTP4 grown under nutrient repletion, about 10% of DW^14^. Under optimal growth conditions, cells shift the carbon flux towards the synthesis of carbohydrates rather than the accumulation of lipids. The latter are predominantly synthesized and accumulated under adverse environmental conditions, such as nutrient depletion. In this context, a two-stage growth system would be able to increase lipid productivities, and thus higher CO_2_ fixation rates^12,14^. In a first stage, cultures could be grown under optimal conditions to reach a high cell concentration, whereas at a later stage lipid induction is achieved *via* environmental stress (e.g. nutrient depletion, high light, salinity, temperature)^12,34,35^.

However, the key strategy to enhance carbon mitigation is the optimization of culture growth. In subtropical or temperate climate zones, seasonal variations of solar irradiance and temperature often lead to impaired microalgal growth during winter^36–40^. Similarly to previous studies^38,39^, the season comparison assay under laboratory conditions (Algem^®^ PBR) revealed that Spring conditions with higher temperatures and light intensities clearly enhance the growth rate and metabolism of *Tetraselmis* sp. CTP4 cultures. An additional enhancement of biomass and lipid productivities and consequently CO_2_ sequestration requires optimization of growth media as well as effective light and CO_2_ delivery into the cultures (the bottleneck of any PBR). In the present work, cultures were grown photoautotrophically, where growth depends on light and inorganic nutrients. However, a mixotrophic growth system that does not rely exclusively on CO_2_ as a carbon source and use organic compounds such as acetic acid or glycerol could improve biomass production as reported for other species^41,42^.

## Conclusions

In conclusion, monoalgal cultures of *Tetraselmis* sp. CTP4 were successfully scaled up to industrial PBR and grown semi-continuously for 60 days without any culture collapse or contamination by a competing microalga. The growth data obtained in the autumn-winter season, demonstrate the robustness of this strain for large-scale production, as well as the interesting biomass productivities that can be obtained under non-optimal environmental conditions. However, as previously discussed, the productivities here presented do not represent the maximum that can be achieved with this microalgal strain. Large-scale production in spring-summer seasons will most probably lead to improved biomass productivity and carbon mitigation, due to the higher microalgal metabolism promoted by increased temperatures and solar radiation.

## Methods

### Microalgae strain and culture medium preparation

All experiments described in the present work were performed at the facilities of CMP (Secil Group, Portugal), between 15^th^ of August and 15^th^ of December 2016. The microalgal strain selected for industrial growth, *Tetraselmis* sp. CTP4, was previously isolated, by the authors, near a wastewater stream in Ria Formosa, in the south of Portugal. The growth characterization under laboratory conditions was published elsewhere^14,15^. All experiments and scale-up were performed with artificial seawater (salinity of 20 g L^−1^) prepared with commercial sodium chloride. Although *Tetraselmis* sp. CTP4 is a euryhaline strain that can withstand wide salt concentrations, the experiments carried out in the present work were performed in at 20 g L^−1^ based on the higher growth performance of cultures previously demonstrated in the laboratory. Guillard’s F2 culture medium adapted to the local water was used in all experiments; cultures were supplemented with the concentrated culture medium to reach a 5-mM concentration of nitrate (70 mg N L^−1^).

### Scale-up of CTP4 cultures

**Figure 7:**
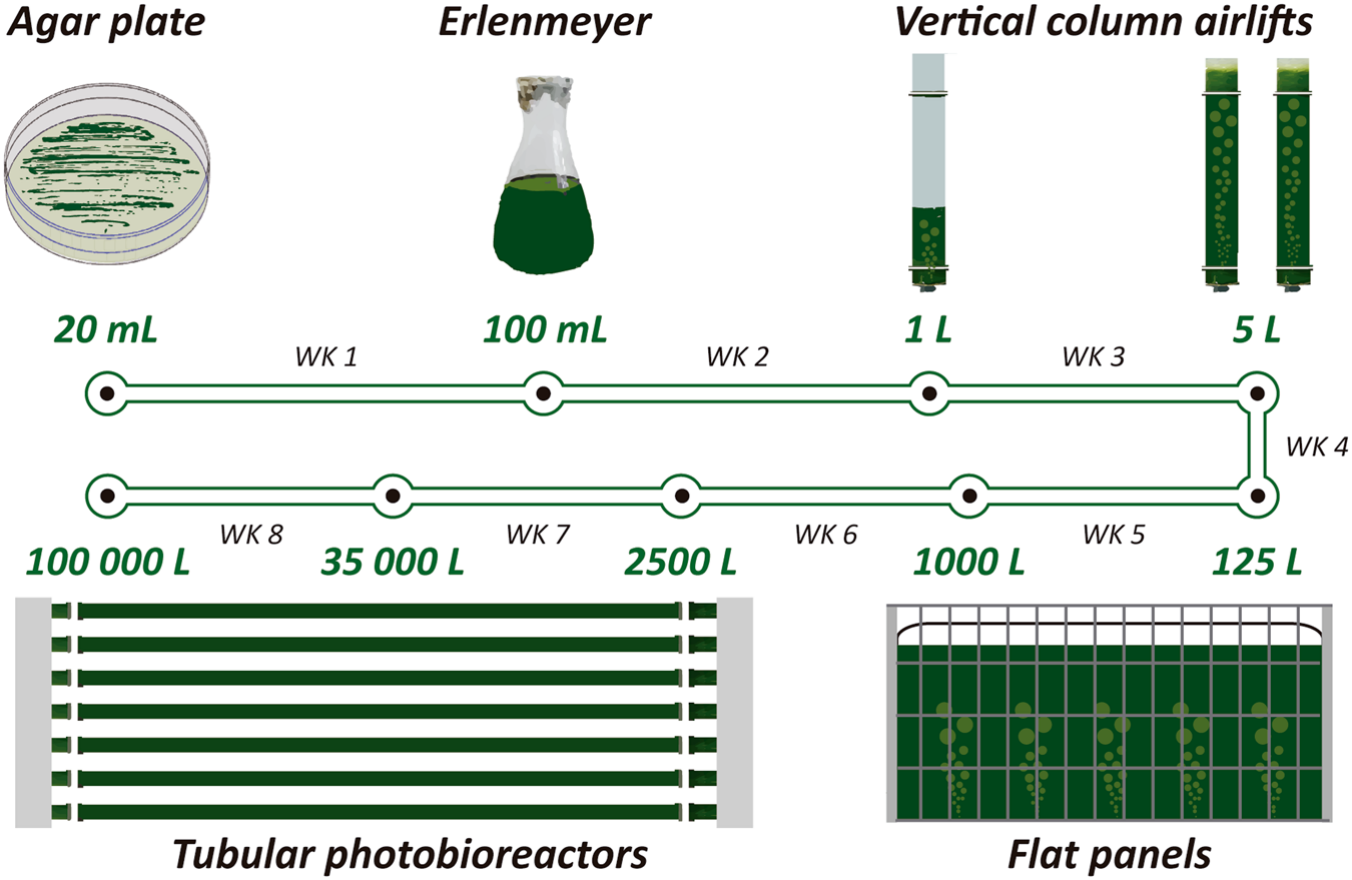
Schematic representation of the scale-up procedure used in the present work. Cultures were transferred every week (WK) to a different production system; the corresponding culture volumes are represented for each system used.

The scale-up procedure (Fig. 7) started with an agar plate (prepared according to Pereira *et al*.^14^) and reached after eight single steps the industrial scale (100 m^3^ PBR). Each scale-up step lasted 7 days as follows: (i) cells were transferred to liquid medium by scrapping algal colonies from the agar plates directly to 100 mL Erlenmeyer flasks that were placed in an orbital shaker under low light intensity (50 µmol photons s^−1^ m^−2^); (ii) and (iii) the 100 mL cultures were inoculated in a vertical 1-L airlift with a 1 L capacity that was subsequently transferred to two 5 L airlifts; (iv) and (v) the cultures obtained in the two 5-L airlifts were used to inoculate a 125-L Flat Panel (FP), which was then used to seed a 1-m^3^ FP (Fig. 1a); (vi) the culture grown in the 1-m^3^ FP was used to inoculate two 2.5-m^3^ pilot-scale tubular PBR (Fig. 1b); (vii) the two pilot-scale PBRs were later used to inoculate an industrial-scale 35-m^3^ tubular PBR; (viii) from this, approximately 30 m^3^ were transferred from the PBR to inoculate the 100-m^3^ tubular PBR (Fig. 1c), while the remaining culture was regrown in the 35-m^3^ PBR upon addition of culture medium.

### Optimization of biomass production

Experiments for the optimization of culture velocity and pH set point for CO_2_ injection were performed in 2.5-m^3^ tubular PBR in duplicates under batch conditions. Fixed culture parameters were chosen according to the results obtained by the previous trials (see Results section for details). Culture velocities of 0.65, 1.01 and 1.35 m s^−1^ were tested at a fixed pH of 8.0, while three distinct pH set points (7.0, 7.5 and 8.0) were tested at a culture velocity of 1.01 m s^−1^. The culture velocity was measured using a Dynasonics DXN (Portable Ultrasonic Measurement System). The pH was adjusted by an automatic CO_2_ injection system (Yokogawa). The local temperature and radiation were registered using a RM Young meteorological station and an Apogee Logan UT SP-110 pyranometer, respectively.

### Industrial production of biomass

The industrial production of microalgae biomass was carried out in 35- and 100-m^3^ horizontal tubular PBR, with an area of implementation of 133 and 405 m^2^, respectively. The photic section of the production system was composed of polymethyl methacrylate (PMMA) tubes (∅_i_ = 56 mm), having a total length and width of 48.2 × 2.5 m and 96.0 × 4.0 m for the 35- and 100-m^3^ PBRs, respectively. The growth trial lasted for 60 days between 17^th^ October and 15^th^ December under a semi-continuous operation. Every 13–14 days, depending on available operational resources, approximately 70% of the total culture volume was harvested while the remaining culture was renewed with fresh growth medium. Both reactors were cultured at a salinity of 20 g L^−1^, with a culture velocity of 1.01 m s^−1^ and a pH set point for CO_2_ injection of 8.0. An in-house system registered the turbidity, pH and temperature inside the PBR in real-time.

### Microscopy

The differential interference contrast (DIC) microscopic images were acquired with the 63 × lenses using a Nikon Eclipse Ni-U and a Zeiss Axioimager Scope A1. Fluorescence microscopy was performed with the Zeiss microscope with the 63 × lenses, using an Axiocam 503 color and Zeiss 64 and 65 HE filter sets. All images were treated with Zen v.2.3 (blue edition) software. Microalgae samples were stained with BODIPY 505/515 as described in Cooper *et al*.^43^ to evaluate the lipid content of the cells.

The presence of contaminants was evaluated by daily microscopic observations of three independent samples in ten microscopic fields. In addition, some samples were analysed by means of flow cytometry corroborating the microscopic results, as described in Schulze *et al*.^15^.

### Growth assessment

Microalgal biomass growth was assessed by means of optical density (OD) and dry weight (DW). The OD of cultures was determined using a Thermo Scientific Genesis 10 S UV-Vis spectrophotometer at a wavelength of 600 and 740 nm. DW was determined by filtering a known volume of culture through 0.45-µm fibreglass filters (VWR). The filter was sequentially washed with the same volume of ammonium formate (35 g L^−1^) and of distilled water. The filters were dried and weighed in AnD MS-70 and Kern DBS 60-3 moisture analysers (120 °C). Ash content was determined by burning 1 g of biomass at 550 °C for 8 hours in a furnace (J. P. Selecta, Sel horn R9-L). A correlation between OD 600 and 740 and AFDW was used to establish the growth curves (previously determined).

### Lipid determination

The total content of lipids in the microalgal biomass was determined using a modified Bligh & Dyer^44^ method previously described in Pereira *et al*.^45^. Briefly, the microalgal pellet was extracted with a mixture of chloroform, methanol and water (2:2:1) using an Ultra-Turrax (IKA) disperser for 2 minutes. Phase separation was achieved by centrifugation for 10 minutes at 3500 *g*; the chloroform phase containing the lipids was removed using a Pasteur pipette and transferred to new vials. A known volume of the lipid extract was then evaporated and the content of lipids was gravimetrically determined.

### CO_2_ sequestration

In order to quantify the CO_2_ mass balances, two rotameters were installed in the 100-m^3^ industrial tubular PBR in the injection valve of the CO_2_ supplying system and in the compressed air valve of the degassing system. To register the outputs of CO_2_ from the PBR (every 5 minutes), a gas analyser (Madur, GA-21 plus) was coupled to the gas exhaust section of the PBR for 30 days (17^th^ Oct – 17^th^ Nov). The CO_2_ mitigation balance was calculated by the sum of CO_2_ supplied by the automatic CO_2_ injection system and the atmospheric CO_2_ introduced from the degasser (compressed air), from which the CO_2_ exhausted from the PBR, as quantified by the gas analyser, was subtracted.

### Elemental analysis and photosynthetic efficiency

Elemental analysis of C, H and N in produced biomass was performed using a Vario el III (Vario EL, Elementar Analyser system, GmbH, Hanau, Germany) according to the procedure provided by the manufacturer.

The higher heating value (HHV; KJ g^−1^) of the biomass produced was calculated according to Callejon-Ferre *et al*.^46^ using the following equation:

$${\rm{H}}{\rm{H}}{\rm{V}}=-3.393+0.507[ \% {\rm{C}}]-0.341[ \% {\rm{H}}]+0.067[ \% {\rm{N}}]$$

where %C, %H and %N represent the carbon, hydrogen and nitrogen content in AFDW, respectively.

PE was calculated by dividing the obtained HHV by the supplied irradiance during a given cultivation interval.

### Algem^®^ photobioreactors season comparison

A season comparison assay was carried out using an Algem^*®*^ PBR (Algenuity, Bedfordshire, UK), in order to assess whether the results obtained outdoors represent the maximum growth that can be obtained with this strain, since the microalga was cultivated in the autumn-winter season. Using the software provided with the equipment, the environmental conditions of Spring and Autumn seasons at the location of AlgaFarm production plant (39.652936 N, −8.988986 W) were simulated. Cultures were mixed at 120 rpm, under constant aeration. CO_2_ was injected automatically using a pH set point of 8.0. The PBR was set to register the optical density at 740 nm every hour.

### Statistical treatment

One-way ANOVA followed by Tukey’s post-hoc test and Analysis of Covariance (ANCOVA) were performed to detect statistical differences between continuous environmental variables (temperature and radiation) and the response variables (volumetric and areal biomass productivities, photosynthetic efficiency and lipid content) using Addinsoft XLSTAT (Version 2016.02.28451). Linear relationships were assessed via a two-tailored Pearson’s test (r). Significance of correlations were tested for using Sigmaplot (Vers. 13, Systat Software Inc.). Significance level for all test was α = 0.05.

## Electronic supplementary material

Supplementary material
