# Supplementary material for: Scale-up and large-scale production of Tetraselmis sp. CTP4 (Chlorophyta) for CO2 mitigation: from an agar plate to 100-m3 industrial photobioreactors
Source: Sci Rep. 2018 Mar 23;8:5112. doi: 10.1038/s41598-018-23340-3 (PMC5865139; doi:10.1038/s41598-018-23340-3)

**Supplementary material**

**Scale-up and large-scale production of *Tetraselmis* sp. CTP4 (Chlorophyta) for CO_2_ mitigation: from an agar plate to 100-m^3^ industrial photobioreactors**

Hugo Pereira^1^, Jaime Paramo^2^, Joana Silva^2^, Ana Marques^2^, Ana Barros^2^, Dinis Mauricio^2^, Tamára Santos^1^, Peter Schulze^3^, Raul Barros^4^, Luísa Gouveia^5^, Luísa Barreira^1^, João Varela^1,*^*.*

^1^ CCMAR - Centre of Marine Sciences, University of Algarve, Gambelas, 8005-139 Faro, Portugal

^2^ CMP - Cimentos Maceira e Pataias, ALGAFARM – Unidade de Produção de Microalgas, 2445 – 411 Pataias, Portugal

^3^ Faculty of Biosciences and Aquaculture, Nord University, 8049 Bodø, Norway

^4^ CIMA - Centro de Investigação Marinha e Ambiental, University of Algarve, Gambelas, 8005-139 Faro, Portugal

^5^ LNEG - Laboratório Nacional de Energia e Geologia, I.P./Bioenergy Unit, Estrada do Paço do Lumiar 22, 1649-038 Lisbon, Portugal

^*^Corresponding author. Tel: +351 289 800 900; Fax: +351 289 800 069

E-mail address: jvarela@ualg.pt

**CO_2_ mitigation calculations**

**Inputs**

**a) CO_2_ injection** (99.99% at 1.5 bar) – Rotameter 20 L min^-1^

Average value = 4870 L day^-1^ (1.5 bar) = 13.20 Kg day^-1^

**b) Degasser** (compressed air; 0.04% at 2.5 bar) – Rotameter = 180 L min^-1^

Average value = 103.68 L day^-1^ (2.5 bar) = 0.47 Kg day^-1^

**Sum of total CO_2_ input (a + b)**

CO_2_ injection + Degasser = 13.67 Kg day^-1^

**Output**

**Exhaust CO_2_ -** CO_2_ detected by gas analyser = 0.3-0.5% - Rotameter = 180 L min^-1^

Exhaust CO_2_ = 777.6-1296 L day^-1^ = 3.51-5.85 Kg day-1

Average value = 1036.8 L day^-1^ = 4.68 Kg day-1

**CO_2_ mitigation (average)**


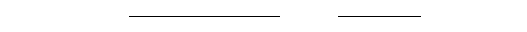


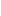


**CO_2_ mitigation (max)** - Exhaust CO_2_ = 0.3%


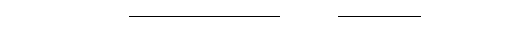


**CO_2_ mitigation (min)** - Exhaust CO_2_ = 0.5%


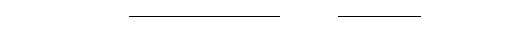

Supplement: Supplementary file 1 — Supplementary material [file 41598_2018_23340_MOESM1_ESM.doc]
